# Clinical outcomes and bleeding events associated with tirofiban combined with intravenous alteplase in acute ischaemic stroke: a retrospective single-center study

**DOI:** 10.3389/fneur.2026.1858221

**Published:** 2026-07-10

**Authors:** Huifang Pang, Hailong Sui, Chunli Fu

**Affiliations:** Department of Neurology, Sinopharm Tongmei General Hospital, Datong, Shanxi, China

**Keywords:** acute ischaemic stroke, alteplase, bleeding risk, intravenous thrombolysis, peri-treatment management, tirofiban

## Abstract

**Objective:**

To evaluate the clinical efficacy and bleeding risk of tirofiban combined with intravenous alteplase in patients with acute ischaemic stroke (AIS).

**Methods:**

This retrospective study identified 90 AIS patients who were divided according to the treatment received into a combined group (CG, *n* = 45; tirofiban plus alteplase) and an alteplase group (AG, *n* = 45; alteplase alone). The 90-day modified Rankin Scale outcome was defined as the primary outcome, whereas neurological deficits, coagulation parameters, inflammatory biomarkers, post-treatment vascular status, activities of daily living, and bleeding events were evaluated as secondary or exploratory outcomes.

**Results:**

Baseline characteristics were comparable between the two groups (*p* > 0.05). NIHSS scores in the CG were significantly lower than in the AG at 24 h, 7 days, and 14 days after treatment (all *p* < 0.01). Post-treatment PT, APTT, and D-dimer were higher in the CG, while fibrinogen was lower (all *p* < 0.001). At day 7, hs-CRP, IL-6, and TNF-*α* were significantly lower in the CG (all p < 0.001). The CG had a higher rate of favorable post-treatment vascular status (*χ*^2^ = 4.731, *p* = 0.030), a higher proportion of favorable 90-day outcomes defined as mRS ≤ 2 (*χ*^2^ = 5.414, *p* = 0.020), and higher Barthel Index scores at day 14 (*p* < 0.001). The total bleeding rate was 28.89% in the CG and 17.78% in the AG, with no statistically significant between-group difference (*χ*^2^ = 1.543, *p* = 0.214).

**Conclusion:**

In this retrospective single-center cohort, tirofiban combined with alteplase was associated with lower NIHSS scores, better post-treatment vascular status, and higher rates of favorable 90-day outcomes, suggesting that combination therapy may potentially improve neurological recovery, attenuate inflammation, and optimize post-treatment vascular status in patients with AIS; however, these findings require confirmation in larger randomized trials.

## 1 Introduction

Acute ischaemic stroke (AIS) is one of the most common neurological emergencies, accounting for approximately 60–80% of all stroke cases, and is associated with persistently high rates of disability and mortality (1, 2). The core pathophysiological mechanism of AIS is ischaemic neuronal necrosis resulting from acute cerebral arterial occlusion; early restoration of cerebral perfusion is therefore fundamental to improving patient outcomes (3). Intravenous alteplase (rt-PA) thrombolysis currently constitutes an important reperfusion strategy for AIS within the therapeutic time window and has been validated across multiple studies for its role in improving functional outcomes. Nevertheless, clinical experience has demonstrated that the vascular recanalization rate achieved with alteplase alone may fall short of expectations, and there remains a risk of post-thrombolytic re-occlusion; consequently, neurological improvement after thrombolysis is suboptimal in a proportion of patients (4, 5).

Tirofiban is a highly selective, non-peptide antagonist of the platelet glycoprotein GPIIb/IIIa receptor that inhibits platelet aggregation and thrombus formation by competitively blocking the binding of fibrinogen to the GPIIb/IIIa receptor (6). In recent years, several studies have explored the feasibility of combining tirofiban with intravenous thrombolysis for AIS, with preliminary findings suggesting that the combination may improve recanalization rates, reduce rethrombosis, and thereby enhance clinical outcomes (7). However, given that tirofiban is an antiplatelet agent, whether its concomitant use on a background of thrombolysis increases the risk of hemorrhagic complications—particularly symptomatic intracranial hemorrhage—has remained a central concern. Published data on the tirofiban–alteplase combination are currently limited by small sample sizes and inconsistent conclusions. Therefore, the differential effects of the two strategies on efficacy and safety warrant further investigation.

In this study, the present retrospective study compared the clinical effects of tirofiban combined with intravenous alteplase versus alteplase alone in patients with AIS, encompassing neurological recovery, coagulation parameter changes, inflammatory biomarker levels, vascular patency status, clinical prognosis, and bleeding risk. The aim was to provide evidence to support therapeutic decision-making and to contribute to the optimization of acute-phase management in patients with AIS.

## 2 Methods

### 2.1 Study design

This was a retrospective comparative study approved by the Ethics Committee of Sinopharm Tongmei General Hospital (approval number: 2024-K-02) and conducted in accordance with the Declaration of Helsinki. Because this was a retrospective observational study based on routine clinical data, the requirement for specific informed consent was waived by the ethics committee. The use of tirofiban in the combined group was determined by the treating physicians after alteplase thrombolysis according to the individual clinical condition of each patient, such as unsatisfactory vascular recanalization or a high risk of re-occlusion; therefore, the treatment groups were not randomly assigned. Patients with AIS admitted to the Department of Neurology of our institution between January 2025 and December 2025 were identified through the hospital information system. This study was reported with reference to the STROBE guidelines.

Inclusion criteria were: (1) diagnosis of AIS fulfilling the American Heart Association/American Stroke Association diagnostic criteria (8) and confirmed by cranial CT or MRI; (2) onset-to-treatment time ≤4.5 h; (3) age ≥18 years; (4) admission NIHSS score (9) of 4–25; and (5) first-ever stroke, or prior stroke without significant residual disability.

Exclusion criteria were: (1) intracranial hemorrhage or subarachnoid hemorrhage; (2) major surgery, head trauma, or stroke within the preceding 3 months; (3) active visceral bleeding or known hemorrhagic diathesis; (4) platelet count <100 × 10⁹/L (applied uniformly to both groups to ensure baseline comparability and reduce bleeding risk); (5) severe hepatic or renal impairment; (6) blood glucose <2.7 mmol/L or >22.2 mmol/L; (7) pregnancy or lactation; and (8) known hypersensitivity to tirofiban or alteplase. Patients who underwent mechanical thrombectomy during the same treatment episode were excluded, and all included patients received intravenous thrombolysis-based treatment alone.

**Figure 1:**
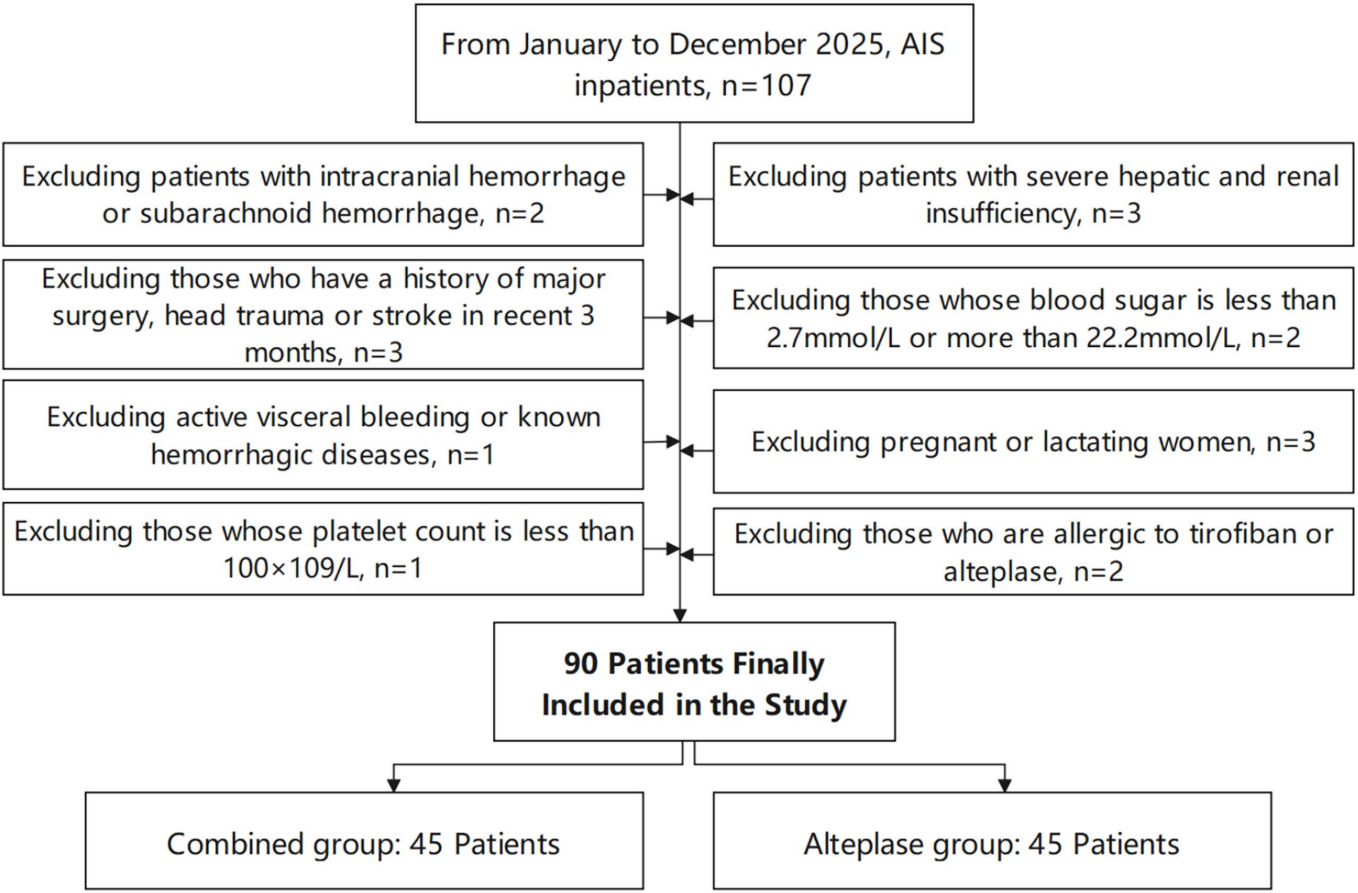
Patient selection flowchart.

After applying these criteria, 90 patients were enrolled. Forty-five patients treated with tirofiban combined with intravenous alteplase were assigned to the combined group (CG), and 45 patients treated with alteplase alone were assigned to the alteplase group (AG). The patient selection process is shown in Figure 1.

### 2.2 Treatment protocols

All patients received standard post-admission workup, vital sign monitoring, and guideline-recommended secondary prevention of stroke.

AG: Intravenous alteplase (rt-PA) was administered at a dose of 0.9 mg/kg (maximum 90 mg); 10% of the total dose was given as an intravenous bolus over 1 min, with the remainder infused continuously over 60 min. Cranial CT was repeated at 24 h post-thrombolysis, and antiplatelet therapy was initiated after hemorrhagic transformation had been excluded.

CG: In addition to the alteplase regimen described above, tirofiban injection (100 mL: 5 mg) was commenced immediately upon completion of alteplase infusion. A loading dose of 0.4 μg/(kg·min) was infused intravenously over 30 min, followed by a maintenance infusion of 0.1 μg/(kg·min) for 24 h; oral antiplatelet therapy was bridged 4 h before tirofiban discontinuation. The tirofiban dosing protocol was informed by published experience in AIS research and adapted to local institutional practice. The loading dose was used to achieve rapid therapeutic plasma concentrations; the entire infusion period was conducted under continuous blood pressure and neurological monitoring.

### 2.3 Outcome measures

#### 2.3.1 Primary outcomes

The primary outcome was 90-day functional prognosis assessed using the modified Rankin Scale (mRS) (10). An mRS score ≤2 was defined as a favorable clinical outcome.

#### 2.3.2 Secondary and exploratory outcomes

Secondary and exploratory outcomes included the following: (1) Neurological deficit scores: the National Institutes of Health Stroke Scale (NIHSS) was used to assess neurological deficits at baseline and at 24 h, 7 days, and 14 days after treatment; (2) Coagulation parameters: prothrombin time (PT), activated partial thromboplastin time (APTT), fibrinogen (FIB), and D-dimer were measured at baseline and at 24 h after treatment; (3) Inflammatory biomarkers: hs-CRP, IL-6, and TNF-*α* were measured at baseline and at 7 days after treatment; (4) activities of daily living: the Barthel Index was used to assess activities of daily living at baseline and at 14 days after treatment; (5) post-treatment vascular status: cranial CT angiography (CTA) or magnetic resonance angiography (MRA) was performed within 24 h after thrombolysis to assess vascular status. CTA was the primary modality, with MRA used as an alternative in patients with contraindications to iodinated contrast agents. The proportions of CTA use were 88.9% in the CG and 86.7% in the AG. Post-treatment vascular status was determined based on angiographic criteria, reflecting vessel status at the time of imaging. Because baseline occlusion data were incomplete, these findings describe vessel status after treatment and do not confirm recanalization. Because of the retrospective design, formal blinded imaging assessment was not performed; and (6) bleeding events: symptomatic intracranial hemorrhage, asymptomatic intracranial hemorrhage, gingival bleeding, skin/mucosal bleeding, and gastrointestinal bleeding were recorded. Platelet count at 24 h after treatment was also reviewed to assess possible tirofiban-associated thrombocytopenia. Symptomatic intracranial hemorrhage was defined using an operational framework informed by ECASS II as neuroimaging evidence of intracranial hemorrhage accompanied by any degree of neurological deterioration, operationally defined as an NIHSS increase of ≥1 point from baseline. It should be noted that this ≥1-point threshold represents a more sensitive operational criterion than the ≥4-point neurological deterioration threshold conventionally employed in ECASS II, SITS-MOST, and other major thrombolysis trials; it is therefore more likely to identify milder neurological deterioration as sICH, and cross-study comparisons of sICH rates should account for this definitional difference. Total bleeding events were tallied by number of affected patients; if a single patient experienced more than one type of bleeding, each type was recorded separately in the corresponding category, but the patient was counted only once in the total bleeding event figure.

### 2.4 Statistical analysis

Statistical analyses were performed using SPSS version 26.0. Continuous variables were assessed for normality using the Shapiro–Wilk test; normally distributed data are expressed as mean ± standard deviation and were compared using the independent-samples *t*-test. Categorical variables are expressed as frequency and percentage [*n* (%)] and were compared using the chi-squared test or Fisher’s exact test, as appropriate (Fisher’s exact test was applied when any expected cell frequency was <5). Repeated-measures data were analyzed using repeated-measures analysis of variance, with time effects, group effects, and time-by-group interaction effects reported. The F statistic, degrees of freedom (df), and effect size (partial *η*^2^) were also reported. Missing longitudinal data were handled using complete-case analysis. A two-sided *p*-value <0.05 was considered statistically significant. Specifically, missing observations were as follows: NIHSS longitudinal data were missing for 2 time-point observations, inflammatory biomarker measurements for 3 observations, and 24-h post-treatment platelet count for 7 patients; all missing data were handled using complete-case analysis, with a negligible impact on the overall conclusions. For the primary outcome (90-day mRS ≤ 2), an exploratory multivariable binary logistic regression analysis (Enter method) was performed with age, sex, baseline NIHSS score, atrial fibrillation, diabetes mellitus, and onset-to-treatment time as covariates. It should be noted that the events-per-variable (EPV) ratio was approximately 8.2 (49 events across 6 covariates), which falls below the recommended threshold of EPV ≥ 10; the regression model therefore carries an increased risk of overfitting and the results should be interpreted as exploratory rather than confirmatory evidence.

## 3 Results

### 3.1 Baseline characteristics

No statistically significant differences were observed between the two groups in sex, age, BMI, onset-to-treatment time, or comorbidities (all *p* > 0.05), indicating good baseline comparability (Table 1). It should be noted, however, that the CG had numerically higher proportions of patients with atrial fibrillation and diabetes mellitus compared with the AG; although neither difference reached statistical significance (*p* > 0.05), these clinical imbalances may represent potential confounders.

**Table 1:** Comparison of baseline characteristics between groups (mean ± SD/[*n* (%)]).

Variable	CG (*n* = 45)	AG (*n* = 45)	*t*/*χ*^2^	*p*-value
Mean age (years)	71.91 ± 6.78	71.58 ± 6.69	0.229	0.820
Male/Female (n)	27/18	20/25	2.178	0.140
BMI (kg/m^2^)	24.58 ± 2.78	24.24 ± 2.63	0.588	0.558
Onset-to-treatment time (h)	2.63 ± 1.15	2.63 ± 1.05	0.010	0.992
Hypertension [*n* (%)]	32 (71.11)	35 (77.78)	0.510	0.475
Diabetes mellitus [*n* (%)]	20 (44.44)	12 (26.67)	3.086	0.079
Dyslipidaemia [*n* (%)]	21 (46.67)	24 (53.33)	0.400	0.527
Atrial fibrillation [*n* (%)]	14 (31.11)	7 (15.56)	2.962	0.085
Admission NIHSS score	13.53 ± 3.42	14.36 ± 3.62	1.102	0.274

CG, combined group (tirofiban plus alteplase); AG, alteplase group (alteplase alone); BMI, body mass index; NIHSS, National Institutes of Health Stroke Scale.

### 3.2 NIHSS scores before and after treatment

**Figure 2:**
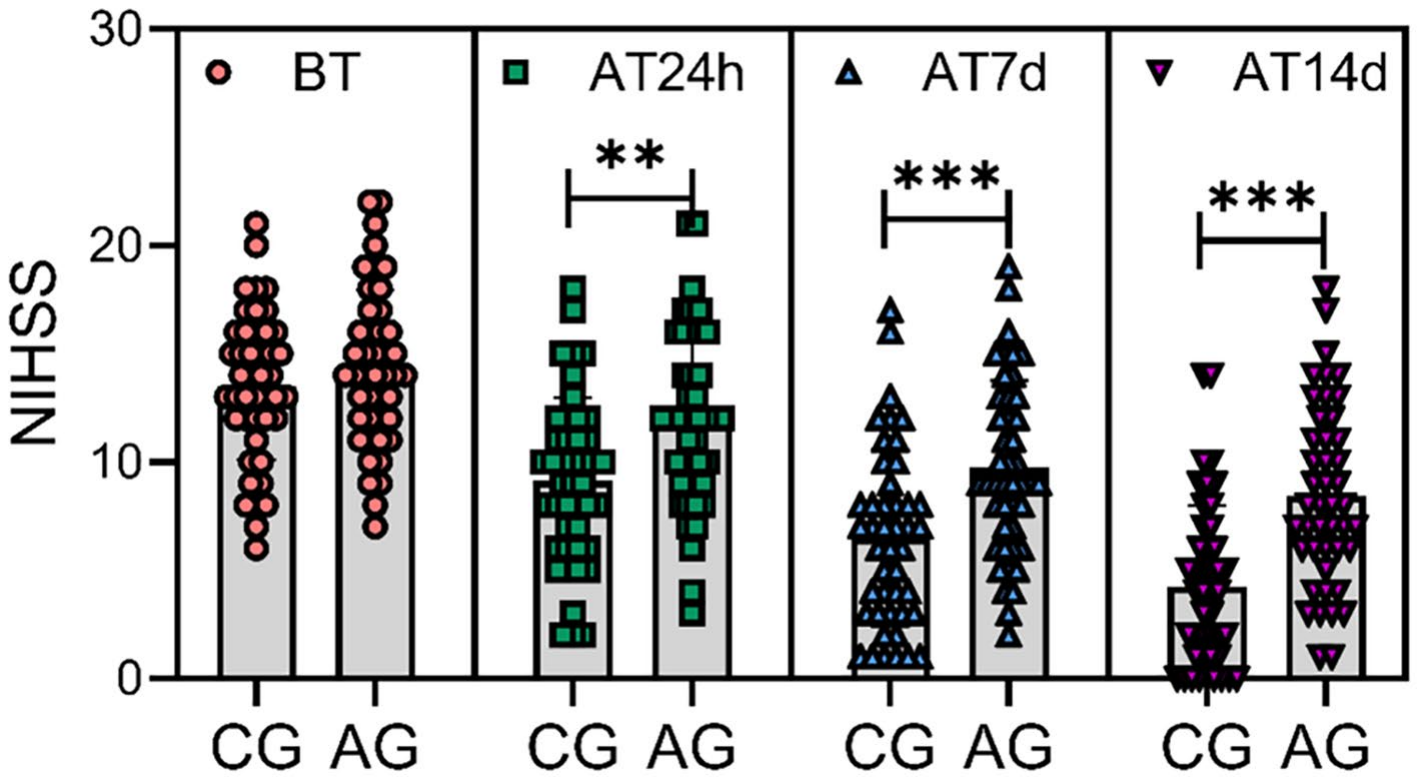
Comparison of NIHSS scores between groups before and after treatment. No significant between-group difference was observed at baseline; at 24 h, 7 days, and 14 days after treatment, NIHSS scores in the CG were significantly lower than in the AG. Repeated-measures ANOVA showed a significant time effect, *F*(3,264) = 89.43, *p* < 0.001, partial *η*^2^ = 0.502; group effect, *F*(1,88) = 12.87, *p* = 0.001, partial *η*^2^ = 0.127; and time-by-group interaction, *F*(3,264) = 4.21, *p* = 0.007, partial *η*^2^ = 0.046. Error bars represent mean ± SD. CG, combined group; AG, alteplase group. **p < 0.01, ***p < 0.001.

No significant difference in baseline NIHSS scores was observed between the two groups (*p* > 0.05). At 24 h, 7 days, and 14 days after treatment, NIHSS scores in the CG were significantly lower than in the AG at all post-treatment time points (all *p* < 0.05). Repeated-measures ANOVA showed significant time effects (*F* = 89.43, df = 3, *p* < 0.001, partial *η*^2^ = 0.502), group effects (*F* = 12.87, df = 1, *p* = 0.001, partial *η*^2^ = 0.127), and time-by-group interaction effects (*F* = 4.21, df = 3, *p* = 0.007, partial *η*^2^ = 0.046). Results are illustrated in Figure 2.

### 3.3 Coagulation parameters before and after treatment

**Figure 3:**
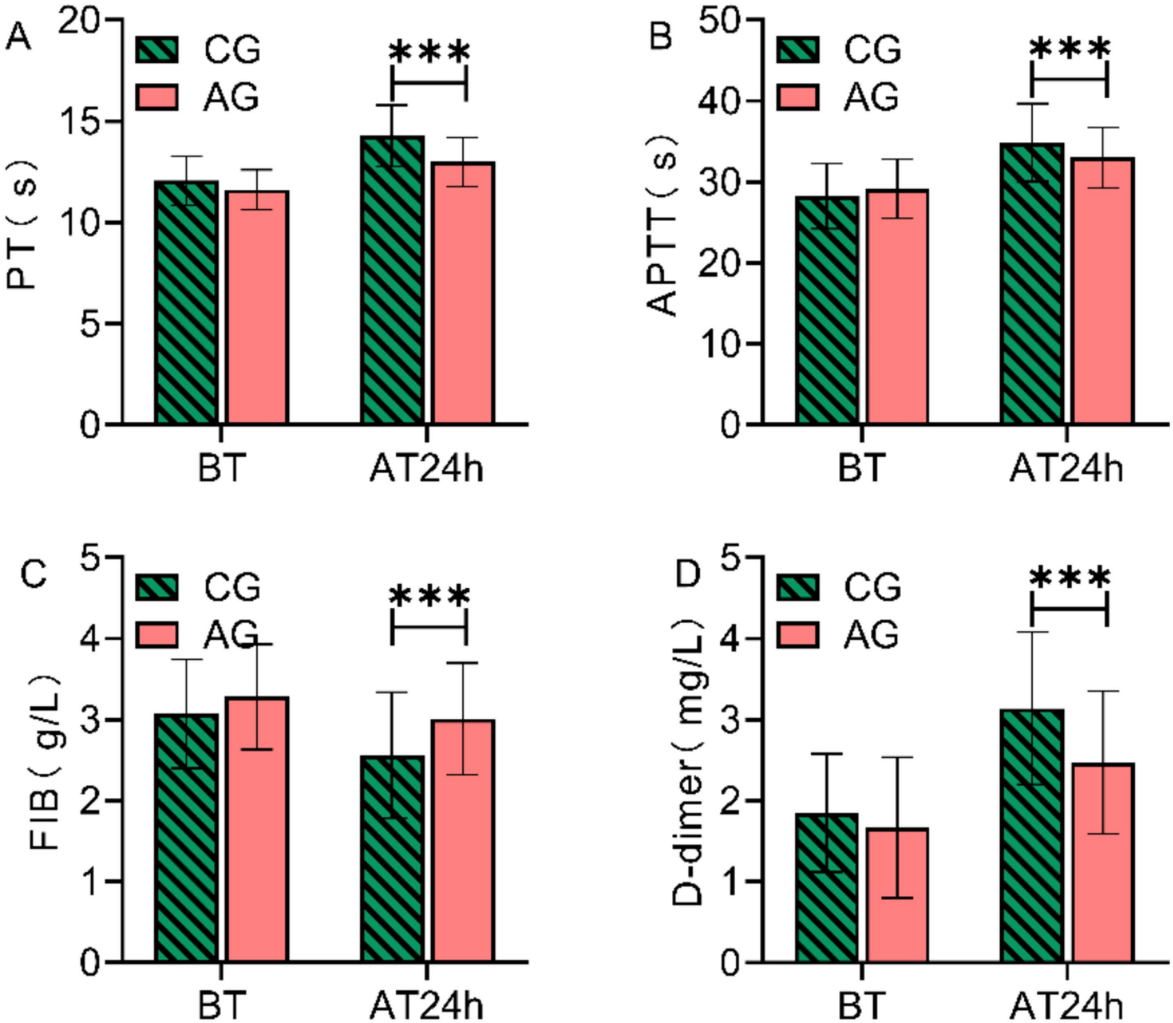
Comparison of coagulation parameters between groups before and after treatment. At 24 h after treatment, PT **(A)**, APTT **(B)**, and D-dimer **(C)** were higher in the CG, whereas FIB **(D)** was lower compared with the AG (all *p* < 0.001). Error bars represent mean ± SD. CG, combined group; AG, alteplase group; PT, prothrombin time; APTT, activated partial thromboplastin time; FIB, fibrinogen. ***p < 0.001.

No significant between-group differences in any coagulation parameter were observed at baseline (all *p* > 0.05). At 24 h after treatment, PT, APTT, and D-dimer were all significantly higher in the CG than in the AG, whereas FIB was significantly lower (all *p* < 0.001). Results are illustrated in Figure 3.

### 3.4 Inflammatory biomarker levels before and after treatment

**Figure 4:**
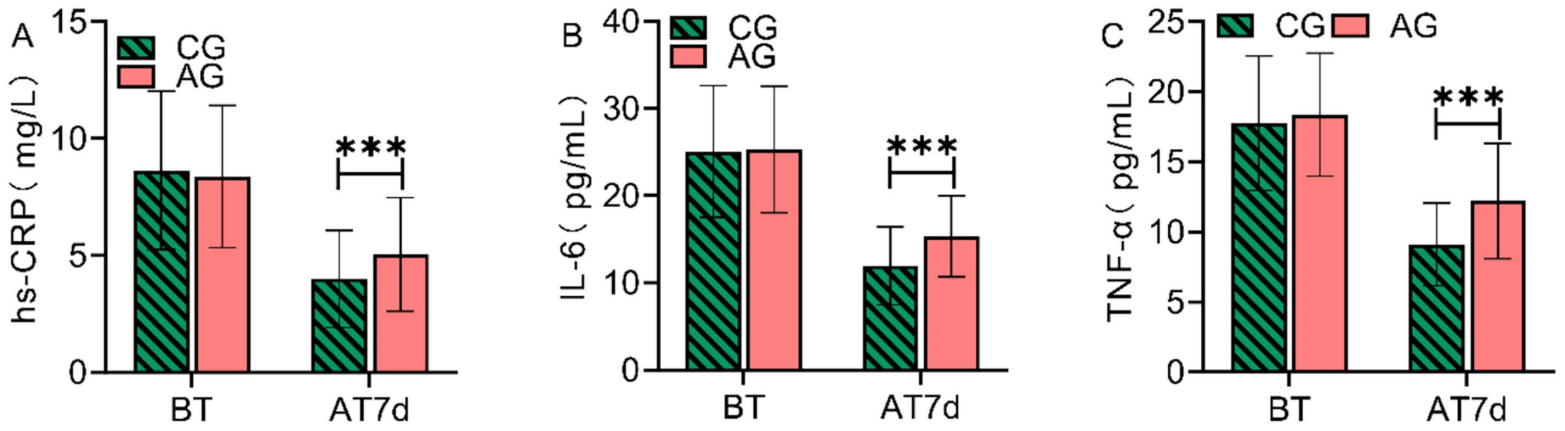
Comparison of inflammatory biomarker levels between groups before and after treatment. At day 7 after treatment, hs-CRP **(A)**, IL-6 **(B)**, and TNF-α **(C)** were all significantly lower in the CG than in the AG (all *p* < 0.001). Error bars represent mean ± SD. CG, combined group; AG, alteplase group; hs-CRP, high-sensitivity C-reactive protein; IL-6, interleukin-6; TNF-α, tumor necrosis factor-α. ***p < 0.001.

No significant between-group differences in baseline inflammatory biomarker levels were observed (all *p* > 0.05). At day 7 after treatment, hs-CRP, IL-6, and TNF-α levels were all significantly lower in the CG than in the AG (all *p* < 0.001). Results are illustrated in Figure 4.

### 3.5 Post-treatment vascular status

The rate of favorable post-treatment vascular status was 73.33% (33/45) in the CG, significantly higher than 51.11% (23/45) in the AG (*χ*^2^ = 4.731, *p* = 0.030; Table 2). Because this was a retrospective study, some patients did not undergo systematic vascular imaging before thrombolysis, and complete baseline occlusion data were not available. Therefore, the 24-h vascular findings should be interpreted as post-treatment vascular status, and the interpretation of vascular patency status is limited by the absence of complete baseline occlusion information.

**Table 2:** Comparison of post-treatment vascular status between groups [*n* (%)].

Group	*n*	Vascular patency [*n* (%)]	No vascular patency [*n* (%)]	*χ*^2^	*P*-value
CG	45	33 (73.33)	12 (26.67)	4.731	0.030
AG	45	23 (51.11)	22 (48.89)		

CG, combined group; AG, alteplase group.

### 3.6 Ninety-day clinical outcomes

**Figure 5:**
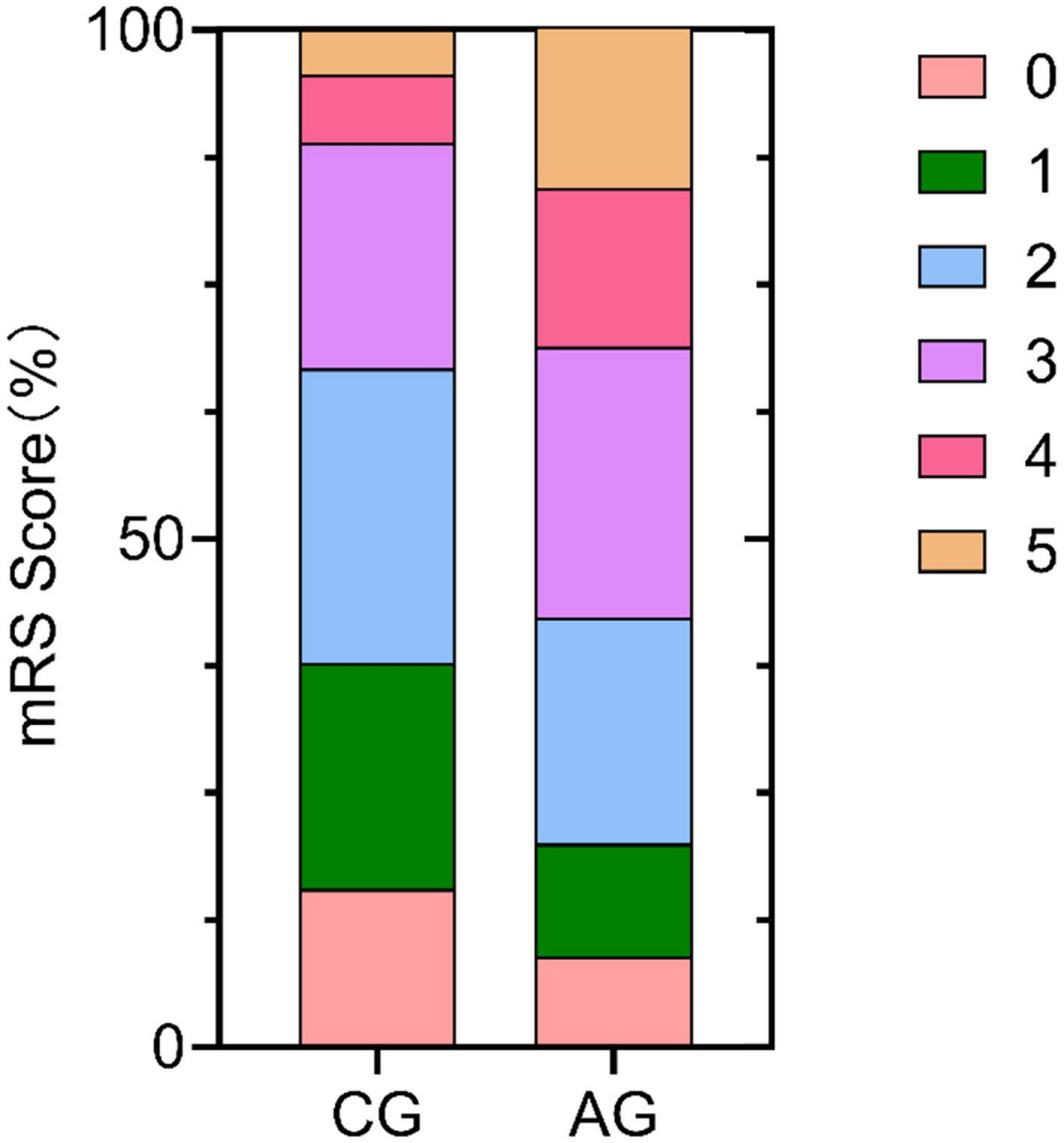
Distribution of 90-day mRS scores between groups.

The proportion of patients with a favorable 90-day outcome (mRS ≤ 2) was 66.67% (30/45) in the CG, significantly higher than 42.22% (19/45) in the AG (*χ*^2^ = 5.414, *p* = 0.020; Table 3 and Figure 5). An exploratory multivariable binary logistic regression analysis was additionally performed, adjusting for age, sex, baseline NIHSS score, atrial fibrillation, diabetes mellitus, and onset-to-treatment time. After adjustment for these potential confounders, the positive association between combination treatment and a favorable 90-day outcome remained consistent in direction (OR = 2.68, 95% CI: 1.01–7.14, *p* = 0.048). However, the EPV ratio was approximately 8.2, falling below the recommended threshold of ≥10; the regression model therefore carries an increased risk of overfitting. These multivariable results should be interpreted as exploratory only and should not substitute for evidence from large-sample randomized controlled trials; the primary conclusion remains based on the unadjusted descriptive analysis.

**Table 3:** Comparison of 90-day clinical outcomes between groups [*n* (%)].

Group	mRS 0	mRS 1	mRS 2	mRS 3	mRS 4	mRS 5	Favorable outcome (mRS ≤ 2) [*n* (%)]	*χ*^2^	*P*-value
CG (*n* = 45)	7 (15.56)	10 (22.22)	13 (28.89)	10 (22.22)	3 (6.67)	2 (4.44)	30 (66.67)	5.414	0.020
AG (*n* = 45)	4 (8.89)	5 (11.11)	10 (22.22)	12 (26.67)	7 (15.56)	7 (15.56)	19 (42.22)		

CG, combined group; AG, alteplase group; mRS, modified Rankin Scale.

### 3.7 Activities of daily living before and after treatment

**Figure 6:**
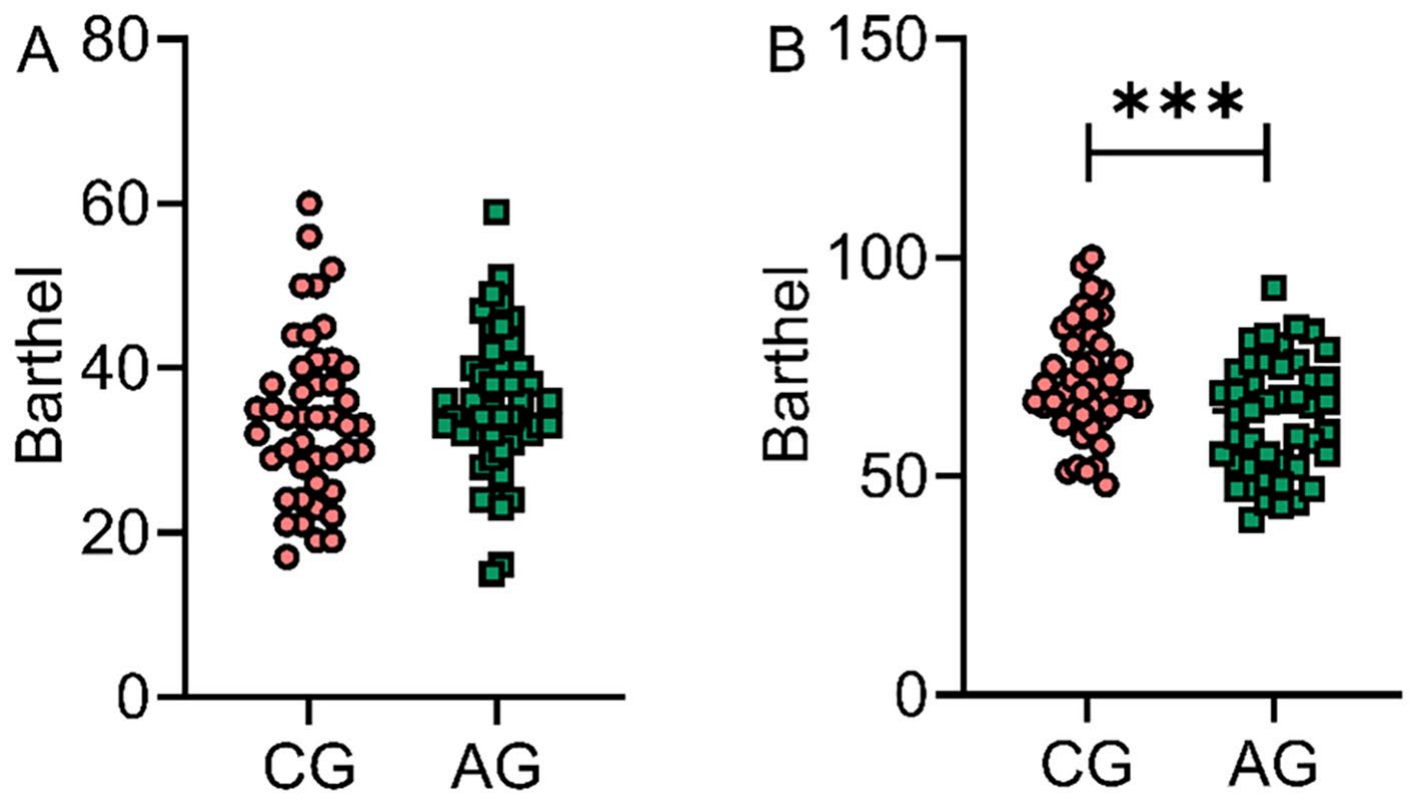
Comparison of activities of daily living between groups before and after treatment. **(A)** Baseline Barthel Index scores. **(B)** Barthel Index scores at day 14 after treatment. At day 14 after treatment, the Barthel Index was significantly higher in the CG than in the AG. Error bars represent mean ± SD. CG, combined group; AG, alteplase group. ***p < 0.001.

No significant between-group difference in baseline Barthel Index scores was observed (*p* > 0.05). At 14 days after treatment, the Barthel Index was significantly higher in the CG than in the AG (*p* < 0.001). Results are illustrated in Figure 6.

### 3.8 Bleeding events

The total bleeding event rate was 28.89% (13/45) in the CG and 17.78% (8/45) in the AG; this difference did not reach statistical significance (*χ*^2^ = 1.543, *p* = 0.214). The rate of symptomatic intracranial hemorrhage also did not differ significantly between groups (*p* > 0.05). The absolute difference in total bleeding rate was approximately 11 percentage points, with a 95% CI of −6.2 to 28.4%. The absolute difference in symptomatic intracranial hemorrhage was 2.22 percentage points, with a 95% CI of −5.6 to 10.1%. It should be noted that the ECASS II-based sICH definition adopted in this study employed a ≥1-point NIHSS worsening threshold, which is considerably more sensitive than the ≥4-point deterioration criterion used in NINDS/SITS-MOST and other major thrombolysis trials. This more sensitive threshold may identify milder degrees of neurological deterioration and could yield a higher apparent sICH rate compared with studies applying the conventional ≥4-point criterion. Caution is therefore warranted when comparing sICH rates across studies; any cross-study comparisons should account for this definitional difference. Detailed results are presented in Table 4.

**Table 4:** Comparison of bleeding event rates between groups [*n* (%)].

Bleeding event	CG (*n* = 45) [*n* (%)]	AG (*n* = 45) [*n* (%)]	Fisher/*χ*^2^	*P*-value
Symptomatic intracranial hemorrhage	2 (4.44)	1 (2.22)	–	1.000
Asymptomatic intracranial hemorrhage	6 (13.33)	3 (6.67)	1.111	0.292
Gingival bleeding	1 (2.22)	1 (2.22)	0.000	1.000
Skin/mucosal bleeding	2 (4.44)	2 (4.44)	0.000	1.000
Gastrointestinal bleeding	2 (4.44)	1 (2.22)	–	1.000
Total bleeding events	13 (28.89)	8 (17.78)	1.543	0.214

CG, combined group; AG, alteplase group. ‘–’ indicates that Fisher’s exact test was applied owing to an expected cell frequency <5. Symptomatic intracranial hemorrhage and gastrointestinal bleeding were analyzed using Fisher’s exact test. If a single patient experienced more than one type of bleeding, each type was recorded separately; however, the patient was counted only once in the total bleeding event figure.

## 4 Discussion

### 4.1 Effects of tirofiban plus alteplase on neurological recovery

The present study demonstrated that NIHSS scores were lower in the CG than in the AG at all post-treatment time points, suggesting that tirofiban combined with intravenous alteplase may contribute to neurological recovery in patients with AIS. This effect may be explained by several mechanisms. Although alteplase mediates thrombus dissolution by converting plasminogen to plasmin and degrading fibrin, thrombin exposed during the thrombolytic process can activate platelets and increase the risk of rethrombosis; tirofiban, by blocking the GPIIb/IIIa receptor, inhibits the final common pathway of platelet aggregation, thereby potentially reducing microthrombus formation and vascular re-occlusion after thrombolysis (11–13). The combination of the two agents may produce a synergistic effect—dissolving existing thrombus while inhibiting new thrombus formation—ultimately improving reperfusion of ischaemic brain tissue and promoting neurological recovery.

### 4.2 Effects of tirofiban plus alteplase on coagulation parameters

The finding that PT, APTT, and D-dimer were higher and FIB was lower in the CG after treatment indicates that combination therapy exerts a more pronounced influence on the coagulation system than alteplase alone. Although tirofiban does not directly interfere with the coagulation cascade, its potent antiplatelet effect may augment the fibrinolytic activity of alteplase, resulting in an overall enhanced antithrombotic effect (14). It should be noted that an elevated D-dimer following combination therapy may partly reflect more extensive fibrinolytic activity; however, D-dimer elevation can also be observed in the context of increased thrombus burden or embolic extension, and its clinical significance should therefore be interpreted in an integrated clinical context rather than unidimensionally attributed to more effective thrombolysis (15, 16). These findings highlight the importance of close monitoring of coagulation parameters during combination therapy to enable early identification of patients at increased bleeding risk and facilitate timely preventive measures.

### 4.3 Effects of tirofiban plus alteplase on inflammatory responses

The CG showed significantly lower levels of hs-CRP, IL-6, and TNF-α at day 7 post-treatment compared with the AG, suggesting that combination therapy may confer advantages in attenuating post-stroke inflammatory responses. These biomarkers were selected because they are commonly used indicators of systemic and neurovascular inflammatory activation after AIS and have been linked to blood–brain barrier injury, infarct progression, and functional prognosis. Following AIS, ischaemia–reperfusion injury is known to trigger intense inflammatory cascades, leading to the release of pro-inflammatory cytokines such as IL-6 and TNF-α, which further amplify cerebral tissue damage (17). In addition to its antiplatelet properties, tirofiban has been reported to possess certain anti-inflammatory effects, potentially by inhibiting the formation of platelet–leucocyte aggregates, thereby reducing leucocyte activation and curtailing the release of inflammatory mediators (6). Furthermore, the better post-treatment vascular status observed with combination therapy may improve cerebral perfusion and help attenuate ischaemia–reperfusion injury and secondary inflammatory responses. These anti-inflammatory effects of the combination regimen may have clinical relevance in terms of reducing cerebral oedema, preserving blood–brain barrier integrity, and facilitating neurological recovery.

### 4.4 Effects of tirofiban plus alteplase on post-treatment vascular status and clinical prognosis

The better post-treatment vascular status and the greater proportion of patients with a favorable 90-day outcome observed in the CG suggest that the combination of tirofiban and alteplase may improve both early vascular status and long-term functional recovery in patients with AIS. Mechanistically, the continuous inhibition of platelet aggregation by tirofiban may help prevent post-thrombolytic vascular re-occlusion and distal microvascular embolization, thereby consolidating the thrombolytic gains; the resulting improvement in vascular status would translate into more complete reperfusion of ischaemic tissue, potentially limiting infarct expansion and reducing penumbral loss (18–20). The significantly higher Barthel Index at day 14 in the CG further supports the advantage of combination therapy in improving activities of daily living.

### 4.5 Bleeding risk analysis

Hemorrhagic complications represent a critical safety consideration in antithrombotic therapy. In the present study, the total bleeding event rate was numerically higher in the CG than in the AG, but the difference did not attain statistical significance; the rates of symptomatic intracranial hemorrhage also did not differ significantly between groups. It should be emphasized, however, that the absolute difference in total bleeding rates between groups was approximately 11 percentage points, and with only 45 patients per group, the statistical power of the present study was limited; a non-significant result does not necessarily indicate the absence of a clinical effect. In this underpowered retrospective cohort, no statistically significant increase in bleeding events was detected with tirofiban plus alteplase. The total bleeding rate was 28.89% versus 17.78%, with an absolute difference of approximately 11 percentage points and a 95% CI of −6.2 to 28.4%; symptomatic intracranial hemorrhage occurred in 4.44% versus 2.22%, with a 95% CI for the difference of −5.6 to 10.1%. These safety findings should therefore be interpreted cautiously and require confirmation in larger randomized controlled trials.

When compared with existing clinical evidence, the present findings are broadly consistent with earlier exploratory safety data on tirofiban in AIS. The SaTIS study provided early evidence that intravenous tirofiban did not markedly increase symptomatic intracranial hemorrhage in AIS (18). The ongoing ASSET-IT trial was designed specifically to evaluate early tirofiban administration after intravenous alteplase thrombolysis, and its intervention strategy is closely related to the treatment approach used in the present study (18). Nevertheless, because the present study was retrospective and small in scale, these comparisons should be regarded as supportive rather than confirmatory.

Several factors may have contributed to the observed safety profile: tirofiban is a short-acting antiplatelet agent with a plasma half-life of approximately 1.5–2 h, and platelet function generally recovers progressively over 4–8 h after discontinuation rather than immediately; in addition, tirofiban was administered at a relatively conservative dose under continuous monitoring throughout the study (21). Notwithstanding these observations, it must be emphasized that clinicians should remain highly vigilant for hemorrhagic complications in practice; for patients at elevated risk—including those of advanced age, with high baseline NIHSS scores, or with pre-existing coagulation abnormalities—the risk–benefit profile of combination therapy should be assessed with particular caution.

### 4.6 Clinical implications and limitations

The present findings suggest that tirofiban combined with intravenous alteplase may offer multiple clinical benefits in AIS, including improved neurological function, reduced systemic inflammation, better post-treatment vascular status, and more favorable prognosis. However, because this was a retrospective single-center study with limited statistical power, the findings should be regarded as exploratory and should not be interpreted as definitive evidence of efficacy or safety. From a clinical perspective, the combination regimen provides a potential approach for enhancing the efficacy of intravenous thrombolysis, and may be particularly relevant for patients with incomplete vascular patency or at risk of re-occlusion.

The study has several limitations. First, it was a single-center retrospective study with a relatively small sample size, limiting the generalisability of the findings. Second, the follow-up period was 90 days, and longer-term outcomes remain unclear. Third, the CG had a numerically higher proportion of patients with atrial fibrillation and diabetes mellitus compared with the AG; although these imbalances did not reach statistical significance, they represent potential confounders that may have influenced efficacy and bleeding outcomes. Fourth, although an exploratory multivariable logistic regression analysis adjusting for atrial fibrillation, diabetes mellitus, baseline NIHSS score, age, sex, and onset-to-treatment time was performed, the events-per-variable ratio was only approximately 8.2—below the recommended threshold of ≥10—and the model therefore carries an increased risk of overfitting; accordingly, these results should be regarded as exploratory and should not replace evidence from large-sample randomized controlled trials. Fifth, the study did not include subgroup analyses stratified by TOAST aetiological subtype or by disabling versus non-disabling stroke status, and differential responses to combination therapy across aetiological subtypes—as well as possible confounding effects of aetiological composition on vascular patency rates and bleeding risk—cannot be excluded. Sixth, a cost-effectiveness analysis was not conducted.

Additional limitations should also be acknowledged. Complete baseline vascular occlusion data were unavailable because some patients did not undergo systematic CTA or MRA before thrombolysis, and baseline occlusion sites were not recorded uniformly; therefore, the 24-h vascular findings reflect post-treatment vascular status rather than confirmed recanalization, and the interpretation of vascular patency status is limited. Imaging assessment was not performed under formal blinded conditions. Baseline ASPECTS scores or infarct volumes, prior antithrombotic therapy, and prior statin use were not collected systematically. In addition, because lymphocyte and monocyte counts were incompletely recorded, the Systemic Inflammatory Response Index could not be calculated. The 24-h platelet count data were also incomplete, preventing a reliable between-group comparison of tirofiban-associated thrombocytopenia. Regarding door-to-needle time, the retrospective information system had limited timestamp precision and some records were incomplete; available data indicated that DNT was within 60 min in both groups, with no apparent between-group difference, but a formal systematic comparison was not feasible. Tirofiban was scheduled to be administered immediately after completion of alteplase infusion; however, the exact median interval from the end of alteplase infusion to the start of tirofiban could not be reliably calculated owing to retrospective recording limitations. Future studies should consider multicenter, large-sample randomized controlled trials to further validate the efficacy and safety of tirofiban combined with intravenous alteplase, and to explore the optimal dose and timing of administration.

## 5 Conclusion

In this retrospective single-center cohort, adjunctive tirofiban following intravenous alteplase thrombolysis was associated with was associated with better neurological recovery, reduced inflammatory biomarker levels, better post-treatment vascular status, and more favorable 90-day functional outcomes in patients with AIS. These associations suggest that the combination may potentially confer clinical benefits; however, they should be interpreted as hypothesis-generating rather than confirmatory evidence. However, no statistically significant increase in bleeding events was detected only within the limits of this underpowered retrospective cohort, and the evidence level remains limited. Larger multicenter randomized controlled trials are required to establish the efficacy and safety of this combination regimen. In clinical practice, strict adherence to therapeutic indications, standardized drug dosing and administration, and close monitoring of coagulation parameters and bleeding signs are essential to ensure the safe use of this regimen.

## Data Availability

The original contributions presented in the study are included in the article/supplementary material, further inquiries can be directed to the corresponding author.

## References

[1] Robba C van Dijk EJ van der Jagt M. Acute ischaemic stroke and its challenges for the intensivist. Eur Heart J Acute Cardiovasc Care. (2022) 11:258–68. doi: 10.1093/ehjacc/zuac004, 35134852

[2] Xu W Guo Y Zhao L Fu R Qin X Zhang Y . The aging immune system: a critical attack on ischemic stroke. Mol Neurobiol. (2025) 62:3322–42. doi: 10.1007/s12035-024-04464-2, 39271626

[3] Arnalich-Montiel A Burgos-Santamaría A Pazó-Sayós L Quintana-Villamandos B. Comprehensive management of stroke: from mechanisms to therapeutic approaches. Int J Mol Sci. (2024) 25:25 (10). doi: 10.3390/ijms25105252, 38791292 PMC11120719

[4] Psychogios K Tsivgoulis G. Intravenous thrombolysis for acute ischemic stroke: why not? Curr Opin Neurol. (2022) 35:10–7. doi: 10.1097/WCO.0000000000001004, 34799512

[5] Mers L Bogdanova A Sekita A Singer L Schmidt M Kallmuenzer B . Safety of tenecteplase versus alteplase for intravenous thrombolysis in acute ischemic stroke patients with direct oral anticoagulation: experience from a German stroke center. Neurol Res Pract. (2025) 7:88. doi: 10.1186/s42466-025-00450-8, 41239434 PMC12619176

[6] Jiang Y Huang W Zhang Y Ji QH. Tirofiban in acute ischemic stroke: mechanistic rationale, clinical advances, and emerging therapeutic strategies. Drugs. (2025) 85:1269–87. doi: 10.1007/s40265-025-02222-9, 40847265 PMC12484258

[7] Lin L Liu F Yi T Zhu Y Yang J Zhao Y . Tirofiban on first-pass recanalization in acute stroke endovascular thrombectomy: the OPTIMISTIC randomized clinical trial. JAMA Netw Open. (2025) 8:e255308. doi: 10.1001/jamanetworkopen.2025.5308, 40244586 PMC12006867

[8] Sacco RL Kasner SE Broderick JP Caplan LR Connors JJ Culebras A . An updated definition of stroke for the 21st century: a statement for healthcare professionals from the American Heart Association/American Stroke Association. Stroke. (2013) 44:2064–89. doi: 10.1161/STR.0b013e318296aeca, 23652265 PMC11078537

[9] Mastrorilli D Mezzetto L D'Oria M Fiorini R Lepidi S Scorsone L . National Institutes of Health stroke scale score at admission can predict functional outcomes in patients with ischemic stroke undergoing carotid endarterectomy. J Vasc Surg. (2022) 75:1661–1669.e2. doi: 10.1016/j.jvs.2021.11.079, 34954269

[10] Saver JL Chaisinanunkul N Campbell BCV Grotta JC Hill MD Khatri P . Standardized nomenclature for modified Rankin scale global disability outcomes: consensus recommendations from stroke therapy academic industry roundtable XI. Stroke. (2021) 52:3054–62. doi: 10.1161/STROKEAHA.121.034480, 34320814

[11] Suzuki Y Mathews NS Sano H Morooka N Honkura N Urano T. Real-time imaging of platelet-initiated plasma clot formation and lysis unveils distinct impacts of anticoagulants. Thromb Haemost. (2025) 125:766–78. doi: 10.1055/a-2497-4213, 39788528 PMC12283144

[12] Zhao Q Yang Y Jin W Chi H Yang D Chen S . Safety and efficacy of ultra-early tirofiban treatment following alteplase in patients with noncardioembolic acute ischemic stroke. J Am Heart Assoc. (2026) 15:e045150. doi: 10.1161/JAHA.125.045150, 41804903

[13] Flora GD Nayak MK Ghatge M Chauhan AK. Metabolic targeting of platelets to combat thrombosis: dawn of a new paradigm? Cardiovasc Res. (2023) 119:2497–507. doi: 10.1093/cvr/cvad149, 37706546 PMC10676458

[14] Muravlev IA Dobrovolsky AB Antonova OA Khaspekova SG Alieva AK Pevzner DV . Effects of antiplatelet drugs on platelet-dependent coagulation reactions. Biomolecules. (2023) 13:1124. doi: 10.3390/biom13071124, 37509160 PMC10377112

[15] Chen J Yang Y Li Y Xu L Zhao C Chen Q . Targeted microbubbles combined with low-power focused ultrasound promote the thrombolysis of acute deep vein thrombosis. Front Bioeng Biotechnol. (2023) 11:1163405. doi: 10.3389/fbioe.2023.1163405, 37008026 PMC10060865

[16] Fair KA Farrell DH McCully BH Rick EA Dewey EN Hilliard C . Fibrinolytic activation in patients with progressive intracranial hemorrhage after traumatic brain injury. J Neurotrauma. (2021) 38:960–6. doi: 10.1089/neu.2018.6234, 31382848 PMC8054516

[17] Pawluk H Woźniak A Tafelska-Kaczmarek A Kosinska A Pawluk M Sergot K . The role of IL-6 in ischemic stroke. Biomolecules. (2025) 15:470. doi: 10.3390/biom15040470, 40305179 PMC12024898

[18] Tao C Liu T Sun J Zhu Y Li R Wang L . Advancing stroke safety and efficacy through early tirofiban administration after intravenous thrombolysis: the multicenter, randomized, placebo-controlled, double-blind ASSET IT trial protocol. Int J Stroke. (2025) 20:373–7. doi: 10.1177/17474930241299666, 39501470

[19] Xiao Z Hu XY Deng LJ Liu JH Liu A. Safety and efficacy of tirofiban versus traditional dual antiplatelet therapy in endovascular treatment of intracranial aneurysms: a systematic review and meta-analysis. J Neurointerv Surg. (2026) 18:755–62. doi: 10.1136/jnis-2024-023021, 40000163

[20] Guo W Xu J Ma L Ma J Li S Ren C . Safety and efficacy of different tirofiban administration routes on acute ischemic stroke patients with successful recanalization: a propensity score matching analysis. CNS Neurosci Ther. (2022) 28:1993–2000. doi: 10.1111/cns.13936, 35962605 PMC9627363

[21] Yang Q He Q Mao X Fan W Luo X. Evaluation of safety and efficacy of tirofiban injection for treating acute ischemic stroke beyond standard time window. Sci Rep. (2025) 15:27399. doi: 10.1038/s41598-025-11882-2, 40721932 PMC12304154

